# Performance evaluation of DEWMA3 in phase-II for capturing changes in simple linear profiles based on run rule mechanism

**DOI:** 10.1038/s41598-023-35779-0

**Published:** 2023-05-27

**Authors:** Rehan Ahmad Khan Sherwani, Humaira Qasim, Shumaila Abbas, Tahir Abbas, Muhammad Aslam

**Affiliations:** 1 College of Statistical and Actuarial Sciences, University of the Punjab, Lahore, Pakistan; 2 Department of Mathematics, College of Sciences, University of Sharjah, Sharjah, UAE; 3 Department of Statistics, Faculty of Science, King Abdulaziz University, Jeddah, 21551 Saudi Arabia

**Keywords:** Engineering, Mathematics and computing

## Abstract

In Statistical Process Control, many techniques exist for monitoring the stability of a process over time. In this work, we study the relationship of the response variable with explanatory variables in the form of linear profiles for detecting changes in slope and intercept of the linear quality profiles. We used the transformation of explanatory variables approach used for make the regression estimates independent of each other to have zero average. A comparative study of three phase-II methods using DEWMA statistics in monitoring and capturing undesirable deviations in the slope, intercept, and variability is also studied by applying different proposed run rules schemes i.e., R_1/1_, R_2/3_, R_3/3_. Monte Carlo simulations were carried out on R-Software for finding the results of proposed schemes by taking various levels of shifts for intercept, slope, and standard deviation in identifying the false alarm rate of a process. The simulation results based on the average run length criterion show that the proposed run rule schemes improve the detection ability of the control structure. Among all the proposed schemes R_2/3_ is found to be the best one because of its quick detection ability of false alarm rate. The proposed scheme also shows superiority in comparison to other schemes. The simulation results are further justified with a real data application.

## Introduction

Total quality management (TQM) refers to the guiding principles for handling, improving, and providing excellent products or services that meet or exceed a consumer's expectations. Quality itself is intangible and strongly relies on the perception of the consumer. Scientific development enables the manufacturing industry to produce a new model of product swiftly over the year that indicates continuous variation in demands from end users. Now the key to competing in a competitive business environment is to produce good quality products with the help of efficient process monitoring techniques by Montgomery^1^. Total quality management is a versatile subject consisting of many subject fields for quality assurance. One of the worth noting fields includes statistical quality control (SQC) which is further classified into two classes i.e., statistical process control (SPC) and acceptance sampling plans (ASP). The SQC uses the processed data in quality control activities which have been collected, analyzed, and interpreted before use. The aim of SQC is to ensure the production of a good quality product that could meet the desires of the consumers in the competitive markets by Oakland^2^. So there arises a need for the most appropriate design procedure for producing a quality product. Customer satisfaction is considered the top priority, which would be gained by delivering defects-free products. Appropriate process designs supported by appropriate tools are always in demand, affirming the requirements of consumers^3,4^.

Shamma & Shamma^5^ developed the double exponentially weighted moving average (DEWMA) statistic, renowned as “Brown′s one‐parameter linear method” in literature for forecasting. The DEWMA chart was proposed as a controlling tool for the monitoring of manufacturing process. For small-to-moderate shifts in the process mean, the DEWMA control chart outperforms the EWMA control chart. However, DEWMA takes both the current and past information into account and has minor variability. Many researchers used DEWMA technique for process monitoring.

Alevizakos et al.^6^ presented a DGWMA (double generally weighted moving averages) control chart for monitoring time between events with a minor time ranging limit. Vicentin et al.^7^ developed a charting technique with a fixed blend of probability distributions. Abbasi et al.^8^ considered the case of random explanatory variable by using DEWMA control charts. Abreu & Schaffer^9^ stated that both “the single and double Exponential Weighted Moving Average control charts” are used by industries to identify small shifts in manufacturing process. Gong et al.^10^ conducted a study on the performance of DEWMA controller to adjust the process when the process points float significantly. Abdella et al.^11^ gave a value addition to the existing literature by extending the implementations of the *m*^*th*^ polynomial profiles in the Phase-II for monitoring by using the DEWMA (double exponentially weighted moving average) statistic. Khanday & Singh^12^ studied the effect of DEWMA model on $\overline{X }$-control chart. They derived the formulae for the scenario where the features of an item possess the implication of DEWMA model. Alkahtani^13^ designed EWMA and DEWMA control charts by taking normality assumption into account.

Many different research trends under the SPC applications have been extensively applied to monitor the process stability over time in different sectors of production. In literature, types of relationship that are considered consists of simple, multiple, and non-linear functions describing the relationship between response variable with one or more process settings rather than using statistical distribution. Kang & Albin^14^ initiated the profiling method by suggesting two different methods i.e. Multivariate T^2^ and EWMA/R for detecting changes in slope and intercept of simple linear quality profiles. Afterwards, the transformation of the explanatory variables to have a zero average for making the regression estimates independent on each other by suggesting three individual EWMA control charts for monitoring the three parameters of simple linear quality profiles i.e. slope, intercept and error-variance were presented by Kim et al.^15^. Monitoring of linear profiles is quite latest with respect to applicability. The linear profile has two phases. In Phase-I previous data is collected for analysing the estimation of numerical constancy of the process disparity. Also, in identifying the “in-control” estimation of the parameters and eliminating the samples associated with any assignable cause of variation and examining the consistency of the process. It is crucial to note the stability of a process which is gained by checking the probability of getting at least one value outside the control limits. In Phase-II various methods can be used having almost different procedures and steps to be followed to achieve the out-of-control values be identified quickly in the manufacturing process from the estimated set up of first phase. Haq et al.^16^ used the individual observations and studied the Phase-II linear profiles through MaxCUSUM, MaxCCUSUM, MaxEWMA, and MaxDEWMA control charts. Haq^17^ also proposed the MEWMA and AMEWMA control charts to assess the simple and multivariate simple linear profiles. Maleki et al.^18^ showed that the in-control and out-of-control states of Phase-II Poisson regression profiles are unfavourably affected by the estimation of the regression parameters. Mahmood et al.^19^ used the standardized residuals of the Poisson regression model and presented the GLM based progressive mean and double progressive mean control chart algorithms for different run rule schemes. Dirbaz et al.^20^ provided some improved versions of phase-II monitoring linear profile schemes by using auxiliary information. Readers interested on some further literature on Phase-I and Phase-II linear profiles may refer^21–27^. More information on this type of control charts can be seen in^28–39^.

Ren et al.^40^ stated that the fault diagnosis with multi-channel profiles is quite challenging as compared to the simple linear profiles. Maleki et al.^41^ studied the “change point estimation of autocorrelated Poisson profiles” by the method of Phase-I monitoring with a condition that there exists autocorrelation among each profile’s explanatory variable. Abbas et al.^42^ introduced Bayesian double exponentially weighted moving average control charts for monitoring the profiles of products and processes. Noorossana et al.^43^ considered the simple linear profiles in Phase-II for analyzing the effect of approximation error.

In linear profile monitoring, run rule schemes are a set of guidelines that help to determine when a process is out of control. These schemes combine multiple run rule to increase the effectiveness of the monitoring process. These run rules help to identify any abnormal patterns or trends in the data that could indicate a process shift. There are not too much literature on the application of run rules for profile monitoring, Riaz and Touqeer^44^ presented a study on run rules schemes and observed the performance of linear profile procedures. The run rule schemes can enhance the sensitivity of the process with different control charting structures. This study considers different run rule schemes designed under DEWMA control charting structure to determine fluctuations in step shifts in all three parameters of profiles model such as intercept (${\beta }_{0}$), slope (${\beta }_{1}$) and error variance. The process parameters are evaluated for different run rule schemes for better detection of possible assignable causes in the process. The rest of the study is organized as follows: the design of new Phase II profiling method for DEWMA is discussed in “Proposed linear profile methodologies”. In “Proposed run rule scheme using DEWMA”, new run rule schemes are proposed using DEWMA. In “Control limits for the proposed schemes”, control limits for proposed schemes are discussed. In “Performance measures”, the average run length characteristics of the proposed scheme are discussed. In “Performance comparison”, a performance evaluation of the proposed scheme is conducted. Discussion of main results is presented in “Discussion”. Concluding remarks are provided in “Case study”.

## Proposed linear profile methodologies

In this section we discussed the Phase II profiling method using DEWMA statistic. Let *Y* be the response variable of a random process having a linear relationship with independent variable *X* i.e.,

1 $$Y_{ij} = \, A_{0} + A_{1} X_{i} + \varepsilon_{ij} , \, j \, = \, 1,2, \ldots , \, i \, = \, 1, \, 2, \, \ldots ,n,$$

where, A_0_ is the in-control parameter of intercept, *A*_1_ is the in-control parameter of slope and ε_ik_ is the random error term that is *i.i.d.* normal (independently and identically normally distributed) with zero mean and variance σ^2^. As we are considering the case of Phase II, the parameters A_0_, A_1_, and σ^2^ used in Eq. (1) are assumed to be known for the in-control values of the above-mentioned parameters. Kang and Albin^14^ monitored the coefficients of regression by using a bivariate-*T*^*2*^ chart which holds the assumption that the *“j”* samples of the ordinary least square estimators of “a_0_” and “a_1_” follows a bivariate normal distribution. Then the OLS estimators are:

2 $${\mathrm{a}}_{0{\rm j}}={\overline{Y} }_{j}-{\mathrm{a}}_{1{\rm j}}{\overline{X} }_{j}\,{\&}\,{\mathrm{a}}_{1{\rm j}}= \frac{{s}_{xy\left(j\right)}}{{s}_{xx}},$$

where,${\overline{Y} }_{j}=\frac{1}{n}\sum_{i=1}^{n}{Y}_{ij }, {\overline{X} }_{j}=\frac{1}{n}\sum_{i=1}^{n}{X}_{ij}$, ${S}_{xy\left(j\right)}=\sum_{i=1}^{n}\left({X}_{i}-\overline{{X }_{j}}\right){Y}_{ij}$, and ${S}_{xx(j)}=\sum_{i=1}^{n}{\left({X}_{i}-{\overline{X} }_{j}\right)}^{2}$. The mean vector of the bivariate normally distributed estimators of ${\mathrm{a}}_{0{\rm j}}$ and ${\mathrm{a}}_{1{ \rm j}}$ is μ = ${\left({\mathrm{a}}_{0}, {\mathrm{a}}_{1}\right)}^{\mathrm{t}}\mathrm{ whereas t}$ he “variance–covariance matrix” is given below:

3 $${\Sigma }_{0}=\left(\begin{array}{cc}{\sigma }_{0}^{2}& {\sigma }_{01}^{2}\\ {\sigma }_{01}^{2}& {\sigma }_{1}^{2}\end{array}\right)$$

Likewise, their variances and covariance will be estimated by using ${\sigma }_{0}^{2}=\left(\frac{{\sigma }^{2}}{n}+\frac{{\overline{\mathrm{x}} }^{2}{\sigma }^{2}}{ {S}_{xx}}\right),$ ${\sigma }_{1}^{2}=\frac{{\sigma }^{2}}{ {S}_{xx}}$ and ${\sigma }_{01}^{2}=-\frac{{\overline{X}\sigma }^{2}}{ {S}_{xx}}$. Kim et al.^15^ made the coefficients of the linear function independent of each other by applying the transformation approach proposed by him. The model is transformed below which is also referred to as KMW method in literature:

Consider model (1) as:

4 $$Y_{ij} = \, A_{0} + A_{1} X_{i} + \varepsilon_{ij} i = \, 1, \, 2, \, \ldots ,n, \, j \, = \, 1, \, 2, \, \ldots ,$$

By adding and subtracting *A*_1_ $\overline{X }$ in Eq. (4) we have:

5 $$\begin{gathered} Y_{ik} = \, A_{0} + A_{1} \overline{X} + A_{1} X_{i} - A_{1} \overline{X} + \varepsilon_{ik} \hfill \\ \Rightarrow Y_{ik} = \, A_{0} + A_{1} \overline{X} + A_{1} (X_{i} - \overline{X} ) + \varepsilon_{ik} \hfill \\ \Rightarrow Y_{ik} = \, B_{0} + B_{1} X_{i}^{*} + \varepsilon_{ij} , \hfill \\ \end{gathered}$$

*where, B*_*0*_ = *A*_*0*_ + *A*_*1*_* ∗ *$\overline{X }$* and B*_*1*_ = *A*_*1*_* and X*_i_^*^ = $({X}_{i}-\overline{X }$*).*

Kim et al.^15^ stated that the mean and variance of the coefficients of the coded model are: ${\mu }_{{b}_{0}}={B}_{0}={A}_{0}+{A}_{1}*\overline{X }$, ${\mu }_{{b}_{1}}={B}_{1}={A}_{1}, {\sigma }_{bo}^{2}=\frac{{\sigma }^{2}}{n}$ and ${\sigma }_{{b}_{1}}^{2}=\frac{{\sigma }^{2}}{{S}_{xx}}$ respectively. Kang and Albin^14^ used the average residuals statistic as an estimator for the error-variance. The residual is calculated by using *e*_ik_ = *y*_ik_ – *B*_0_ − *B*_1_ *X*_i_^*^ and its average is $\overline{{e }_{k}}=\frac{\sum_{i=1}^{n}{e}_{i\dot{k}}}{n}$ where *“n”* is used for developing the *k*th profile and represents the number of independent variables.

The DEWMA statistic for the intercept, slope, and error are as follows:

DEWMA for intercept:

$${E}_{k}^{\left({b}_{0}\right)}={\lambda }_{1}^{\left({b}_{0}\right)}{b}_{0k}+\left(1-{\lambda }_{1}^{\left({b}_{0}\right)}\right){E}_{k-1}^{\left(bo\right)}$$

6 $${D}_{k}^{\left({b}_{0}\right)}={\lambda }_{2}^{\left({b}_{0}\right)}{E}_{k}^{\left({b}_{0}\right)}+\left(1-{\lambda }_{2}^{\left({b}_{0}\right)}\right){D}_{k-1}^{\left(bo\right)}, \mathrm{k}= 1, 2,$$

DEWMA for slope:

$${E}_{k}^{\left({b}_{1}\right)}={\lambda }_{1}^{\left({b}_{1}\right)}{b}_{1k}+\left(1-{\lambda }_{1}^{\left({b}_{1}\right)}\right){E}_{k-1}^{\left({b}_{1}\right)}$$

7 $${D}_{k}^{\left({b}_{1}\right)}={\lambda }_{2}^{\left({b}_{1}\right)}{E}_{k}^{\left({b}_{1}\right)}+\left(1-{\lambda }_{2}^{\left({b}_{1}\right)}\right){D}_{k-1}^{\left({b}_{1}\right)}, \mathrm{k}= 1, 2,$$

DEWMA for error variance:

$${E}_{k}^{\left(e\right)}=max \{{\lambda }_{1}^{\left(e\right)} (lnMSEk) + (1- {\lambda }_{1}^{\left(e\right)}) {E}_{k-1}^{\left(e\right)}, ln\upsigma 20\},$$

8 $${D}_{k}^{\left(e\right)}= max \{{\lambda }_{2}^{\left(e\right)} (lnMSEk) + (1- {\lambda }_{2}^{\left(e\right)}) {E}_{k-1}^{\left(e\right)}, ln\sigma 20\}, \mathrm{k}= 1, 2,$$

where, the initial values for ${E}_{j}^{\left({b}_{0}\right)}, {D}_{j}^{\left({b}_{0}\right)}, {E}_{j}^{\left({b}_{1}\right)}\,and\,{D}_{j}^{\left({b}_{1}\right)}$ are set as zero and ${\lambda }_{1}^{\left({b}_{0}\right)}, {\lambda }_{2}^{\left({b}_{0}\right)} , {\lambda }_{1}^{\left({b}_{1}\right)},{\lambda }_{2}^{\left({b}_{1}\right)}$ are smoothing parameters and λ_1_ > 0, λ_2_ ≤ 1. This research study will only consider the case where λ_1_ = λ_2_ = λ. The value of λ = 0.2 is taken for comparison purpose. The exact variances of the intercept, slope and error variance are stated below:

9 $${\sigma }_{exact}^{2\left({b}_{0}\right)}=\frac{{\left({\lambda }^{\left({b}_{0}\right)}\right)}^{4}\left[1+{\left({\forall }^{\left({b}_{0}\right)}\right)}^{2}-\left({k}^{2}+2k+1\right){\left({\forall }^{\left({b}_{0}\right)}\right)}^{2k}+\left(2{k}^{2}+2k-1\right){\left({\forall }^{\left({b}_{0}\right)}\right)}^{2k+2}-{k}^{2}{\left({\forall }^{\left({b}_{0}\right)}\right)}^{2k+4}\right]{\sigma }_{bo}^{2}}{{\left(1-{\left({\forall }^{\left({b}_{0}\right)}\right)}^{2}\right)}^{3}}$$

10 $${\sigma }_{exact}^{2\left({b}_{1}\right)}=\frac{{\left({\lambda }^{\left({b}_{1}\right)}\right)}^{4}[1+{\left({\forall }^{\left({b}_{1}\right)}\right)}^{2}-\left({k}^{2}+2k+1\right){\left({\forall }^{\left({b}_{1}\right)}\right)}^{2k}+\left(2{k}^{2}+2k-1\right){\left({\forall }^{\left({b}_{1}\right)}\right)}^{2k+2}-{k}^{2}{\left({\forall }^{\left({b}_{1}\right)}\right)}^{2k+4} ]{ \sigma }_{b1}^{2}}{{\left(1-{\left({\forall }^{\left({b}_{1}\right)}\right)}^{2}\right)}^{3}}$$

11 $${\sigma }_{exact}^{2\left(e\right)}=\frac{{\left({\lambda }^{\left(e\right)}\right)}^{4}[1+{\left({\forall }^{\left(e\right)}\right)}^{2}-\left({k}^{2}+2k+1\right){\left({\forall }^{\left(\left(e\right)\right)}\right)}^{2k}+\left(2{k}^{2}+2k-1\right){\left({\forall }^{\left(e\right)}\right)}^{2k+2}-{k}^{2}{\left({\forall }^{\left(e\right)}\right)}^{2k+4} ]{ \sigma }_{e}^{2}}{{\left(1-{\left({\forall }^{\left(e\right)}\right)}^{2}\right)}^{3}}$$

where, $${\forall }^{\left({b}_{0}\right)}$$=$$(1-{\lambda }^{\left({b}_{0}\right)})$$, $${\forall }^{\left({b}_{1}\right)}$$=$$(1-{\lambda }^{\left({b}_{1}\right)}){, \forall }^{\left(e\right)}$$=$$(1-{\lambda }^{\left(e\right)}), {\sigma }_{bo}^{2}=\frac{{\sigma }^{2}}{n}$$, $${\sigma }_{{b}_{1}}^{2}=\frac{{\sigma }^{2}}{{S}_{xx}}$$ and $${\sigma }_{e}^{2}=\frac{{\sigma }^{2}}{n}$$.

Mahmoud and Woodall^45^ discussed about the derivation of exact variances and their control limits in detail. The three DEWMA control charts are presented by Alkahtani^13^ along with its exact control limits by using the equation of control limit i.e., ${h}_{ik}={\mu }_{0}\pm {L}_{i}{\sigma }_{exact} ,$ where *i* = 1,2,3, …… and *k* is the profile number. The value of *L*_*i*_ is found to satisfy a certain false alarm rate.

## Proposed run rule scheme using DEWMA

In this section, we propose three run rule schemes along with its structures using DEWMA statistic. The suitable names assigned to the proposed schemes include DEWMA_1/1_ or R_1/1_ scheme, DEWMA_2/3_ or R_2/3_ scheme and DEWMA_3/3_ or R_3/3_ scheme. These schemes are introduced to increase the detection performance “for small to moderate shifts in the process.”

### DEWMA_1/1_ or R_1/1_ scheme

In DEWMA_1/1_ scheme, the fraction one by one represents that if any one point is plotted either above the upper signaling limits or below the lower signaling limits then the process will be considered as out-of-control.

### DEWMA_2/3_ or R_2/3_ scheme

In DEWMA_2/3_ scheme, the fraction two by three represents that if any two consecutive points out of three points falls on either below the lower signaling limits or above the upper signaling limits then the process will be considered as out-of-control. There is no restriction imposed on the third point, so it may lie between the upper and lower control limits.

### DEWMA_3/3_ or R_3/3_ scheme

In DEWMA_3/3_ scheme, the fraction three by three represents that if any three consecutive points falls either below the lower signaling limits or above the upper signaling limits then the process will be considered as out-of-control.

## Control limits for the proposed schemes

The term signaling limits used above in all the four proposed schemes are just like the simple control limits, but the difference is created here only to elaborate these limits for intercept, slope and error separately. These limits are specified by average run length (ARL).

For intercept: The process will give out-of-control signal when this condition is violated:

${D}_{k}^{({b}_{0})}$ < *LCL*_*a*_ or ${D}_{k}^{({b}_{0})}$> *UCL*_*a*_ and the control limits for intercept of the proposed schemes is set as under:

12 $$LCLa={\upmu }_{b0} - La\sqrt{{\sigma }_{exact}^{2\left({b}_{0}\right)}} ; UCLa = {\upmu }_{b0} + La\sqrt{{\sigma }_{exact}^{2\left({b}_{0}\right)}}$$

For slope: The process will give out-of-control signal when this condition is not met:

${D}_{k}^{({b}_{1})}$ < *LCL*_*b*_ or ${D}_{k}^{({b}_{1})}$> *UCL*_*b*_ and the control limits for slope of the proposed schemes is set as under:

13 $$LCLb={\mu }_{b1}-Lb\sqrt{{\sigma }_{exact}^{2\left({b}_{1}\right)}} ; \quad UCLb ={\mu }_{b1} + Lb\sqrt{{\sigma }_{exact}^{2\left({b}_{1}\right)}}$$

For error variance: The process will give out-of-control signal when this condition is violated:

${D}_{k}^{(e)}$ < *LCL*_*e*_ or ${D}_{k}^{(e)}$ > *UCL*_*e*_ and the control limits for slope of the proposed schemes is set as under:

14 $$LCLe ={\mu }_{e}-Le\sqrt{{\sigma }_{exact}^{2\left(e\right)}} ; \quad UCLe ={\mu }_{e} + Le\sqrt{{\sigma }_{exact}^{2\left(e\right)}}$$

## Performance measures

The control charting structure defined above is evaluated using the different individual performance measures such as: average run length (ARL), standard deviation of run length (SDRL), median of run length (MDRL) and coefficient of variation of run length (CVRL). The detailed description of these measures is given as follows:

### Average run length

The *ARL* values can be defined as the average number of points must occur before a point signifies an out-of-control signal. For the Shewhart type control charts if the measurements are uncorrelated, the *ARL* can be calculated as: $ARL= \frac{1}{p}$. Where *p* can be defined as the probability of a point overreaches the control limits. The ARL values are categorized as in-control *ARL (ARL*_*0*_*)* and out-of-control *ARL *(*ARL*_*1*_)^46^.

### Standard deviation of run length

The SDRL is the measure of divergence of the run length and denoted by SDRL_0_ under in-control situation and denoted by SDRL_1_ when the process mean departs (an out-of-control state). The SDRL is an individual performance used to evaluate the dispersion in the run length distribution. It is an additional indicator and is used to examine the distributional spread of run length^47^.

### Median of run length

On similar lines the MDRL is used to compute the center point of the run length distribution. The median of run length (MDRL) is also an important individual evaluation measure for control chart used to access the skewness of the RL performance. The MDRL values are categorized as in-control *MDRL (MDRL*_*0*_*)* and out-of-control *MDRL (MDRL*_*1*_*)*.

### Coefficient of variation of run length

The CVRL is a new measure introduced in this article to compute the coefficient of variation property of run length distribution. The coefficient of variation of run length (CVRL) is a statistical quality control tool used to assess the stability of a production process. The CVRL measures the variability in the number of units produced between consecutive runs that are in statistical control. The CVRL is computed as the ratio of the standard deviation of the run length (the number of units produced between two consecutive runs) to the mean run length, expressed as a percentage.

The CVRL criterion is used to determine whether a production process is in statistical control, meaning that it is stable and producing products with consistent quality. A production process is in control if the CVRL is less than or equal to a specified threshold value. The threshold value is determined based on the desired level of quality, the cost of producing non-conforming units, and other factors. To compute the CVRL, the following steps can be followed:

- Collect data on the number of units produced between consecutive runs (i.e., the run length) for a certain period.
- Calculate the mean run length, which is the average number of units produced between consecutive runs.
- Calculate the standard deviation of the run length, which measures the variability in the number of units produced between consecutive runs.
- Compute the CVRL as the ratio of the standard deviation of the run length to the mean run length, expressed as a percentage:
  $${\text{CVRL}}\, = \,\left( {{\text{Standard deviation of run length}}{/}{\text{Mean run length}}} \right)\, \times \,{1}00\%$$

Compare the computed CVRL value to the specified threshold value to determine whether the production process is in statistical control.

If the computed CVRL value is less than or equal to the threshold value, the production process is in-control. If the computed CVRL value is greater than the threshold value, the production process is out-of-control, and corrective action may be necessary to bring it back into control. The CVRL values are categorized as in-control *CVRL (CVRL*_*0*_*)* and out-of-control *CVRL (CVRL*_*1*_*) *^48–50^.

## Performance comparison

The zero state ARL performance of DEWMA3 is compared by using different run rule schemes with the existing profiling methods that are found in the paper of Abdella et al.^46^. He made a comparative study by using Hoteling *T*^2^, DEWMA3, and EWMA/R initially proposed by Kang and Albin^14^ and Kim et al.^15^. Abdella et al.^51^ proposed a set of methods for the monitoring of profile model in Phase-II after assuming in-control process parameter values. The in-control situation described as: ${Y}_{i}=3+2{X}_{i+}{\in }_{ij}$ where *X* is fixed i.e., *X* = {2, 4, 6, 8} and ${\epsilon }_{ij}\sim N\left(0,{\sigma }^{2}=1\right)$. In this article, we have used the transformed profile model as: ${Y}_{ij}=13+2{X}_{i}^{*}+{\epsilon }_{ij}$. The control limit coefficients are adjusted for individual ARL_0_ of around 600 for process parameters of DEWMA charts which provided the overall ARL_0_ of roughly 200. As we have three process parameters to be studied. For each process parameter we get an individual ARL_0_ and then the overall ARL_0_ is found by the expression: $\frac{1}{overall {ARL}_{0}}=\frac{1}{Individual\,{ARL}_{01}}+\frac{1}{Individual\,{ARL}_{02}}+\frac{1}{Individual\,{ARL}_{03}}.$ Abdella et al.^51^, Kang and Albin^14^ and Kim et al.^15^ considered three different types of step shifts i.e., in the intercept, slope and error variance of the regression line that are also used in this study for comparison purpose after using R-Software based on 10,000 simulations.

## Simulation study

The performance of the proposed schemes will be evaluated on the basis of comparison with the study made by Abdella et al.^51^. They compared the results produced by using DEWMA3 versus existing charting techniques like Hotelling’s T^2^, EWMA/R and dMEWMA and showed that the performance of DEWMA3 was quite better among existing techniques. Now, we will be using DEWMA3 in this study and will apply different run rule schemes on DEWMA3 i.e., for intercept, slope and error term separately for the evaluation of statistical performance of the proposed schemes. We will also consider ARL under wide range of shift levels for fair comparison and for drawing beneficial conclusions.

The smoothing constant of $\lambda =0.2$ is considered for the computation of overall in-control ARL = 200 and individual in-control ARL = 600 for each scenario. Basically $\lambda$ is a smoothing constant which is used in the statistic of DEWMA and EWMA control charts and gives weights to the past information. We compared the two scenarios with the existing scheme separately. An appropriate value of the pre-specified coefficients L_a_, L_b_ and L_e_ were considered for each setup. The subscripts represent the pre-specified value of *L* for intercept, slope, and error respectively. By setting the individual in-control ARL for intercept, slope and standard deviation, the value of ARL = 200 was set at approximately 600 for individual in-control ARL’s of intercept, slope, and standard deviation. R_1/1_, R_2/3_, R_3/3_ represented the proposed classical DEWMA3 structure for run rules scheme. Similarly, **Ωa, Ωb** and **Ωe** represent the changes made in step shifts of intercept, slope, and error respectively. All the above-mentioned notations have been used in tables and figures.

For first scheme R_1/1_, results are presented in Tables 1, 2, 3 (for intercept, slope, and error variance respectively). The values for shifting factor “Ω_a_” was set at 0, 0.2, 0.3, 0.4, 0.5, 0.6, 0.8, 1.0, 1.5, 2.0, 2.5 and 3.0 under the designed structures of DEWMA3 for all run rule schemes.

**Table 1:** ARL, SDRL, MDRL and CVRL under shift in intercept from A_0_ to A_0_ + Ω_a_ σ by using classical 1 by 1 run rule for $\lambda$ = 0.2.

Ω_a_	L_a_ = 2.7395, $\lambda$ = 0.2			
	ARL	SDRL	MDRL	CVRL
0.0	200.12	207.76	134.00	103.80
0.2	43.98	41.62	32.00	94.63
0.3	20.43	16.89	16.00	82.71
0.4	12.22	8.99	10.00	73.49
0.5	8.27	5.62	7.00	67.85
0.6	6.01	3.77	5.00	62.69
0.8	3.82	2.27	3.00	59.35
1.0	2.67	1.50	2.00	56.24
1.5	1.47	0.66	1.00	45.04
2.0	1.10	0.31	1.00	28.53
2.5	1.01	0.11	1.00	10.84
3.0	1.00	0.03	1.00	2.83

**Table 2:** ARL, SDRL, MDRL and CVRL under shift in slope from A_1_ to A_1_ + Ω_b_ σ by using classical 1 by 1 run rule for $\lambda$ = 0.2.

Ω_b_	L_b_ = 2.7263, $\lambda$ = 0.2			
	ARL	SDRL	MDRL	CVRL
0	200.01	207.61	134.00	103.80
0.03	62.61	61.42	44.00	98.11
0.04	39.19	36.85	29.00	94.02
0.05	26.38	22.81	20.00	86.49
0.075	12.64	9.15	11.00	72.39
0.1	7.72	5.11	7.00	66.26
0.15	4.04	2.39	4.00	59.21
0.2	2.52	1.41	2.00	56.01
0.25	1.82	0.91	2.00	50.25
0.3	1.42	0.63	1.00	43.99
0.35	1.21	0.44	1.00	36.27

**Table 3:** ARL, SDRL, CVRL and MDRL under shift in error term from A_e_ to Ω_E_ σ by using classical 1 by 1 run rule for $\lambda$ = 0.2.

Ωe	L_e_ = 2.7640, $\lambda$ = 0.2			
	ARL	SDRL	MDRL	CVRL
1	200.61	208.19	134.00	103.77
1.2	30.10	33.93	19.00	112.71
1.4	9.28	10.66	5.00	114.83
1.6	4.63	5.14	3.00	110.86
1.8	2.98	3.06	2.00	102.43
2	2.28	2.21	1.00	96.66
2.4	1.55	1.20	1.00	77.35
2.6	1.39	0.92	1.00	66.03
2.8	1.29	0.76	1.00	58.80
3	1.22	0.64	1.00	52.68
3.5	1.12	0.41	1.00	36.76

For second scheme R_2/3_, results are presented Tables 4, 5, 6 (for intercept, slope, and error variance respectively). The values for shifting factor “Ω_b_” was set at 0, 0.03, 0.04, 0.05, 0.075, 0.10, 0.15, 0.20, 0.25, 0.30 and 0.35 under the designed structures of DEWMA3 for all run rule schemes. For third and final stage, Tables 7, 8, 9 of intercept, slope, and error variance respectively. The values for shifting factor “Ω_e_” was set at 1, 1.2, 1.4, 1.6, 1.8, 2.0, 2.4, 2.6, 2.8, 3.0 and 3.5 under the designed structures of DEWMA3 for all run rule schemes.

**Table 4:** ARL, SDRL, MDRL and CVRL under shift in intercept from A_0_ to A_0_ + Ω_a_ σ by using classical 2 by 3 run rule for $\lambda$ = 0.2.

Ω_a_	L_a_ = 2.3700, $\lambda$ = 0.2			
	ARL	SDRL	MDRL	CVRL
0.0	200.48	203.64	138.00	101.57
0.2	33.31	29.37	25.00	88.17
0.3	17.16	13.41	14.00	78.12
0.4	10.78	7.21	9.00	66.82
0.5	7.79	4.69	7.00	60.15
0.6	6.03	3.37	5.00	55.76
0.8	4.14	1.97	4.00	47.58
1.0	3.21	1.29	3.00	40.18
1.5	2.31	0.56	2.00	24.26
2.0	2.04	0.22	2.00	10.73
2.5	2.01	0.07	2.00	3.48
3.0	2.00	0.01	2.00	0.71

**Table 5:** ARL, SDRL, MDRL and CVRL under shift in slope from A_1_ to A_1_ + Ω_b_ σ by using classical 2 by 3 run rule for $\lambda$ = 0.2.

Ω_b_	L_b_ = 2.3775, $\lambda$ = 0.2			
	ARL	SDRL	MDRL	CVRL
0	200.92	203.91	138.00	101.49
0.03	43.89	40.47	32.00	92.20
0.04	28.55	24.56	22.00	86.04
0.05	20.28	16.64	16.00	82.04
0.075	10.94	7.38	9.00	67.43
0.1	7.23	4.26	6.00	58.96
0.15	4.20	1.99	4.00	47.52
0.2	3.07	1.18	3.00	38.42
0.25	2.53	0.76	2.00	30.04
0.3	2.24	0.48	2.00	21.55
0.35	2.10	0.31	2.00	15.01

**Table 6:** ARL, SDRL, CVRL and MDRL under shift in error term from A_e_ to Ω_E_ σ by using classical 2 by 3 run rule for $\lambda$ = 0.2.

Ωe	L_e_ = 2.5650, $\lambda$ = 0.2			
	ARL	SDRL	MDRL	CVRL
1	200.55	203.45	138.00	101.44
1.2	35.45	36.88	24.00	104.04
1.4	12.25	11.84	8.00	96.62
1.6	6.97	6.14	5.00	87.95
1.8	4.78	3.75	3.00	78.57
2	3.84	2.72	3.00	70.92
2.4	2.89	1.58	2.00	54.70
2.6	2.67	1.28	2.00	47.84
2.8	2.52	1.09	2.00	43.22
3	2.42	0.97	2.00	40.27
3.5	2.25	0.66	2.00	29.55

**Table 7:** ARL, SDRL, MDRL and CVRL under shift in intercept from A_0_ to A_0_ + Ω_a_ σ by using classical 3 by 3 run rule for $\lambda$ = 0.2.

Ω_a_	L_a_ = 2.2690, $\lambda$ = 0.2			
	ARL	SDRL	MDRL	CVRL
0.0	200.62	200.87	138.00	100.13
0.2	33.97	28.78	26.00	84.71
0.3	17.85	12.98	14.00	72.70
0.4	11.52	6.97	10.00	60.50
0.5	8.59	4.66	8.00	54.23
0.6	6.85	3.25	6.00	47.42
0.8	5.03	1.91	5.00	37.90
1.0	4.11	1.25	4.00	30.43
1.5	3.25	0.51	3.00	15.65
2.0	3.04	0.20	3.00	6.61
2.5	3.01	0.06	3.00	1.88
3.0	3.00	0.01	3.00	0.47

**Table 8:** ARL, SDRL, MDRL and CVRL under shift in slope from A_1_ to A_1_ + Ω_b_ σ by using classical 3 by 3 run rule for $\lambda$ = 0.2.

Ω_b_	L_b_ = 2.2690, $\lambda$ = 0.2			
	ARL	SDRL	MDRL	CVRL
0	200.62	200.87	138.00	100.13
0.03	44.11	39.25	32.00	88.99
0.04	29.12	24.29	22.00	83.43
0.05	20.87	16.24	17.00	77.80
0.075	11.67	7.21	10.00	61.74
0.1	8.01	4.12	7.00	51.38
0.15	5.08	1.98	5.00	39.08
0.2	3.98	1.15	4.00	28.86
0.25	3.46	0.71	3.00	20.60
0.3	3.20	0.46	3.00	14.28
0.35	3.08	0.29	3.00	9.34

**Table 9:** ARL, SDRL, CVRL and MDRL under shift in error term from A_e_ to Ω_E_ σ by using classical 3 by 3 run rule for $\lambda$ = 0.2.

Ωe	L_e_ = 2.3900, $\lambda$ = 0.2			
	ARL	SDRL	MDRL	CVRL
1	199.97	200.39	138.00	100.21
1.2	37.45	36.90	26.00	98.54
1.4	13.83	11.97	10.00	86.57
1.6	8.09	6.13	6.00	75.76
1.8	5.95	3.85	5.00	64.82
2	4.88	2.68	4.00	55.01
2.4	3.96	1.71	3.00	43.19
2.6	3.70	1.36	3.00	36.92
2.8	3.54	1.16	3.00	32.98
3	3.41	0.97	3.00	28.61
3.5	3.24	0.69	3.00	21.29

## Graphical analysis

**Figure 1:**
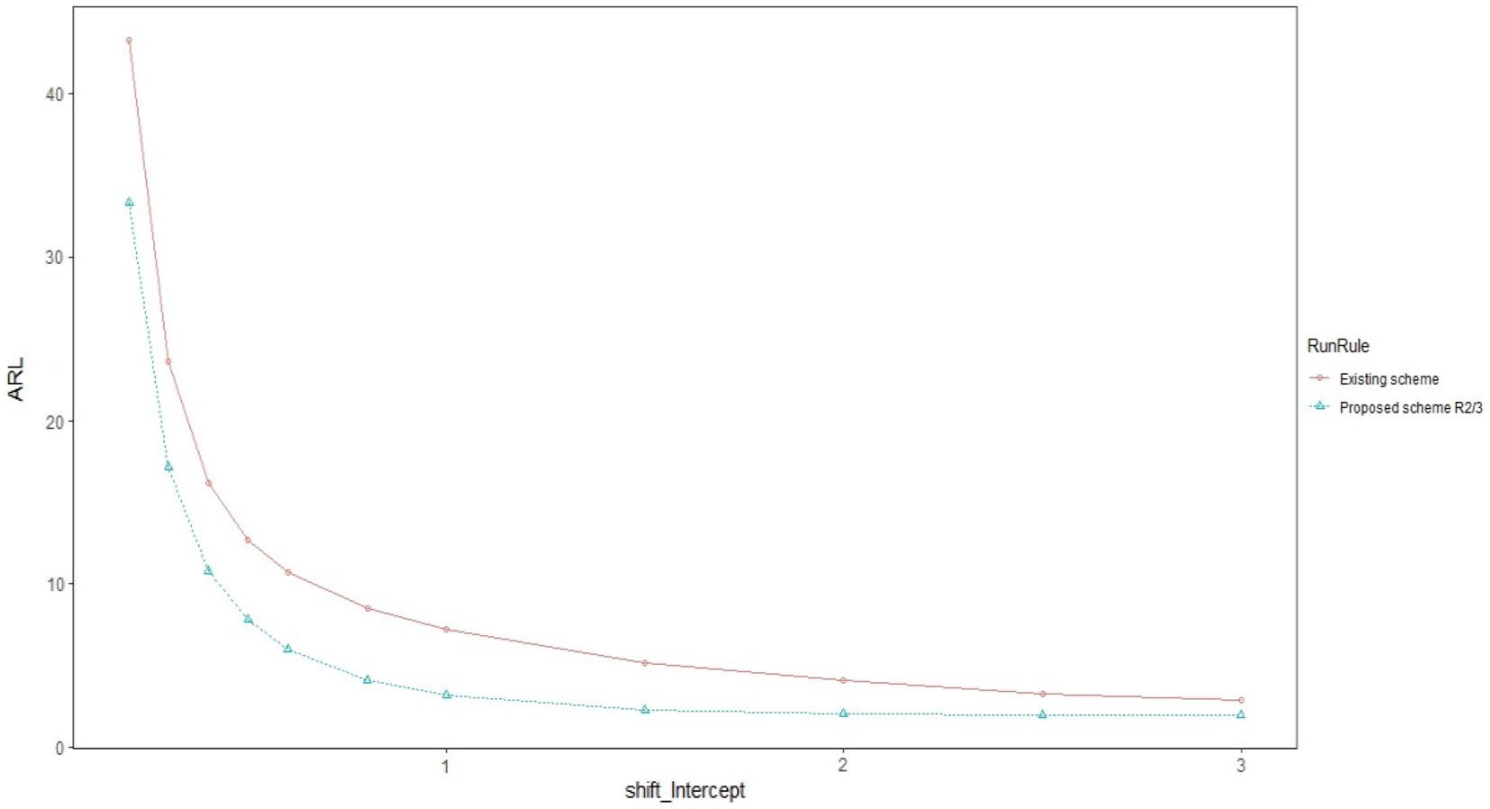
ARL curve under the best proposed scheme-II & the existing scheme under Ω_a_ at ARL = 200 with ${\varvec{\lambda}}$ = 0.2.

**Figure 2:**
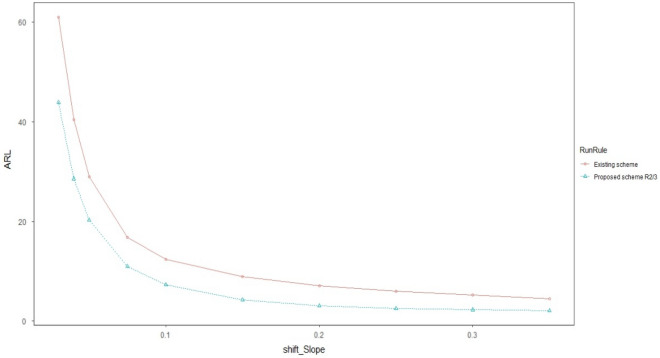
ARL curve under the best proposed scheme-II & the existing scheme under Ω_b_ at ARL = 200 with ${\varvec{\lambda}}$ = 0.2.

**Figure 3:**
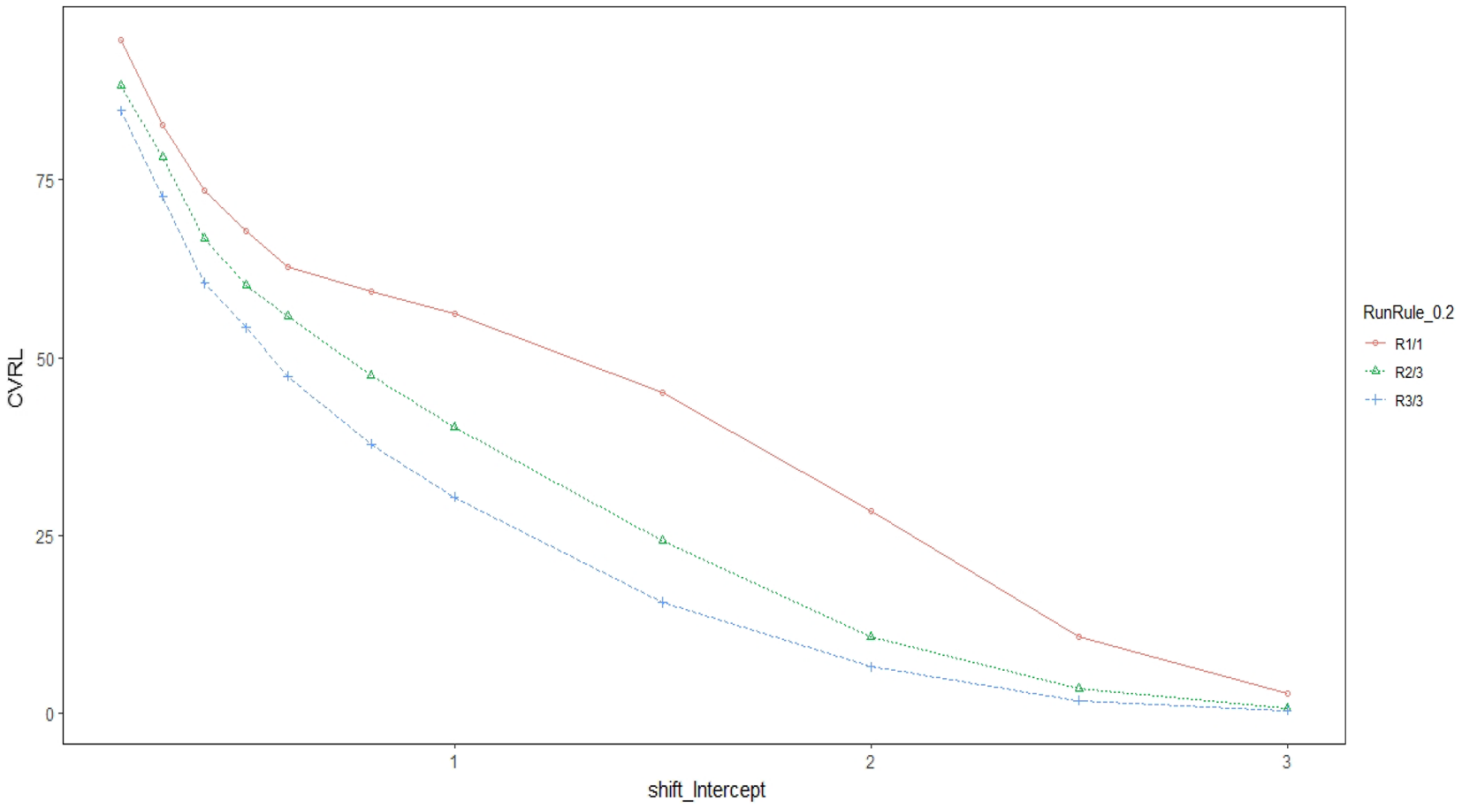
CVRL curve under all proposed schemes-I, II & III under Ω_a_ at ARL = 200 with ${\varvec{\lambda}}$ = 0.2.

**Figure 4:**
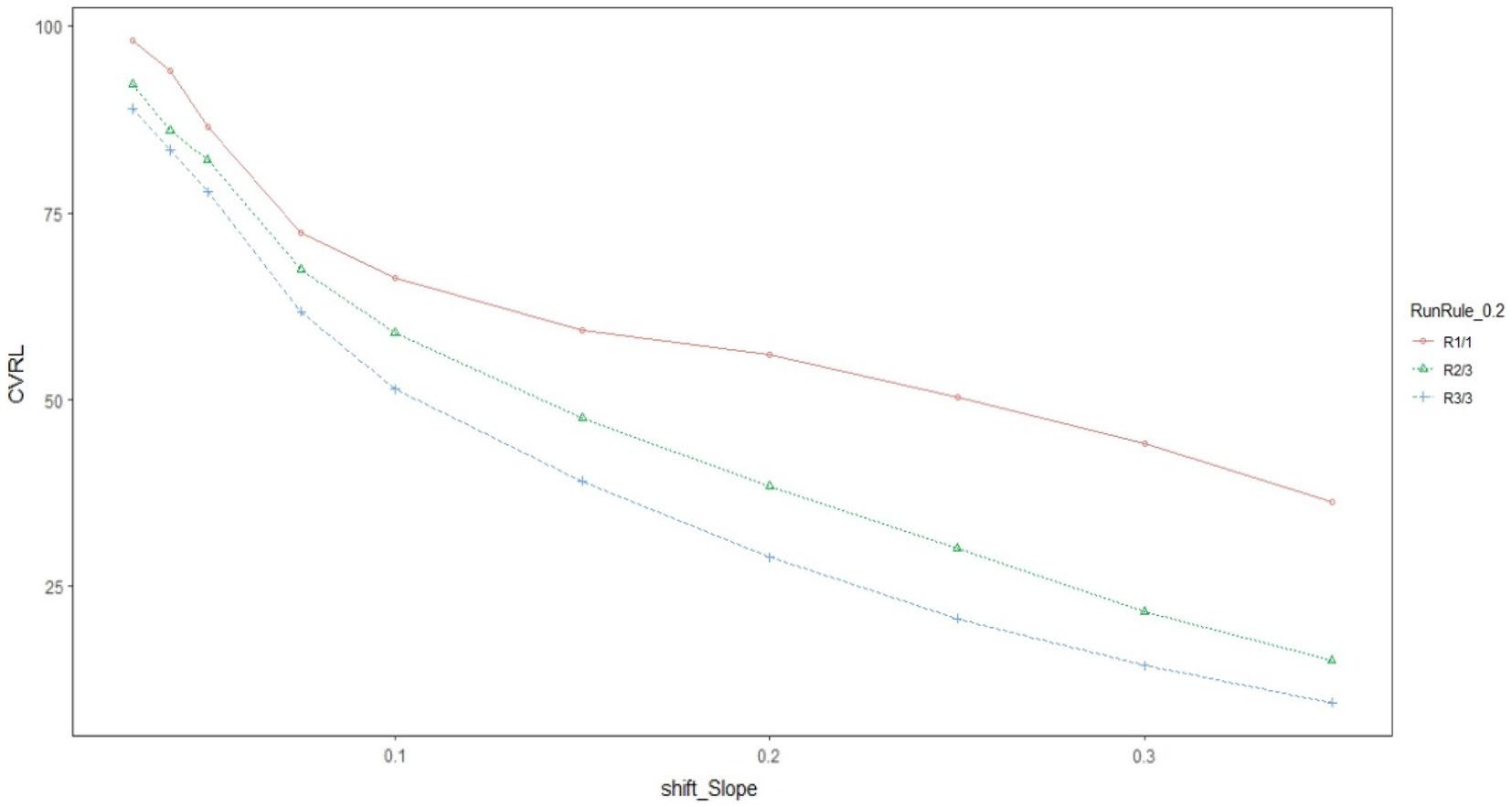
CVRL curve under all proposed schemes-I, II & III under Ω_b_ at ARL = 200 with ${\varvec{\lambda}}$ = 0.2.

**Figure 5:**
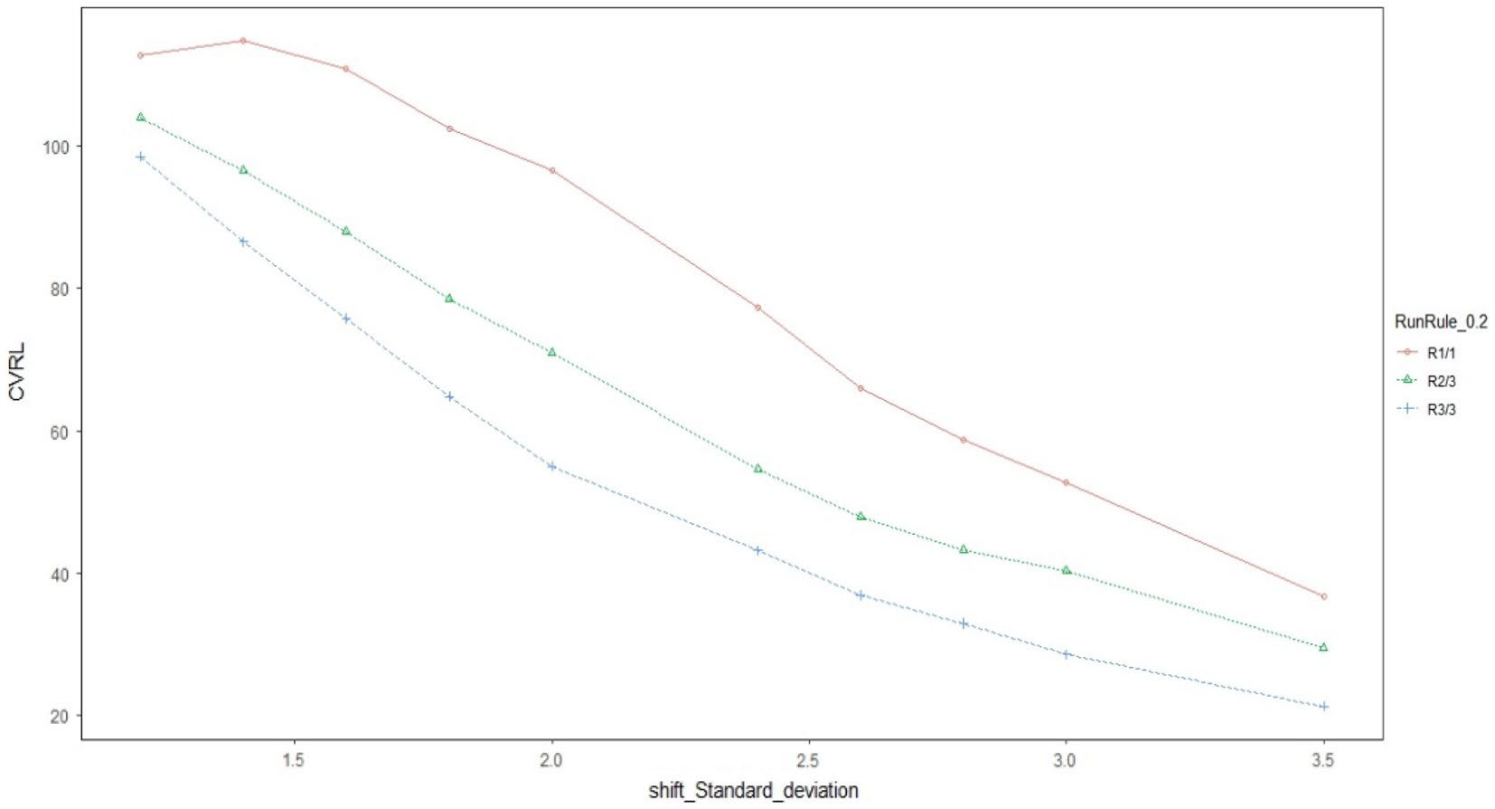
CVRL curve under all proposed schemes-I, II & III under Ω_e_ at ARL = 200 with ${\varvec{\lambda}}$ = 0.2.

**Figure 6:**
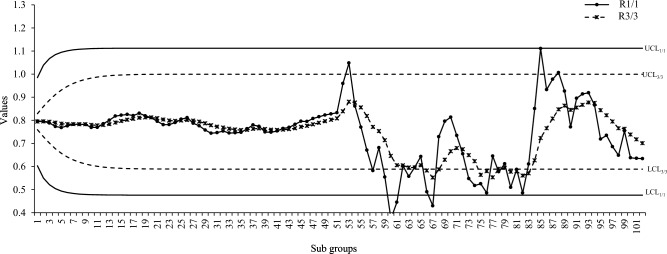
DEWMA chart display under R_1/1_ and R_3/3_ run rule scheme for shift in intercept.

Graphical comparison of all the three proposed schemes is made with each other to get a visual display of their performance. The ARL and CVRL measures of the different proposed schemes corresponding to the shifts Ω_a_, Ω_b_, and Ω_e_ will be shown through graphs_._ We have created graphs for the various levels of intercept shift (Ω_a_), slope shift (Ω_b_) and error variance shift (Ω_e_) against each proposed scheme i.e., R_1/1,_ R_2/3_ and R_3/3_ as shown in Figs. 1 and 2 for assessing the performance through ARL measure of proposed charts by using smoothing constant of $\lambda =0.2$ to check which smoothing constant yields the better result against the other for checking the performance of proposed charts. We compared the efficiency of our best proposed chart i.e. R_2/3_ against the existing scheme given by Abdella et al.^51^ under Ω_a_ and Ω_b_ for $\lambda =0.2$ only because they derived their results by using the value of smoothing constant as 0.2 which is shown in Figs. 1 and 2. We also used CVRL measure for comparing the performance of proposed charts on various shift levels of Ω_a_, Ω_b_ and Ω_e_ by using ${\varvec{\lambda}}=0.2$ as presented in Figs. 3, 4 and 5. The DEWMA chart display under R_1/1_ and R_3/3_ run rule scheme in Fig. 6.

## Discussion

With the help of simulation study, the results revealed that $\lambda$ = 0.1 is giving excellent results than $\lambda$ = 0.2 for detecting the false alarm rate in all the three cases discussed above i.e., in R_1/1_, R_2/3_, R_3/3_. The results reported here only for $\lambda$ = 0.2 and a comparative study can be presented on request. Similarly, difference was observed in the results produced by evaluating the performance of the proposed charts by using ARL’s of both the smoothing constants i.e., $\lambda$ = 0.1 and $\lambda$ = 0.2 but both are showing that all the three proposed schemes work more efficiently for identification of small changes in shift levels than the results produced by the existing study of Abdella et. al^51^ shown in Tables 1, 2, 3, 4, 5, 6, 7, 8 and 9.

Tables 1, 4 and 7 showing results for the purposed schemes for intercept showing that R_1/1_ works better than existing scheme and R_2/3_ works even better than R_1/1_ in term of comparing their ARL and R_3/3_ performs better than R_1/1_. The same case exists while considering slope into account that can be seen in Table 2, 5 and 8. This is the case when the value of smoothing constant is taken as 0.2.

From Tables 1, 2, 3, 4, 5, 6, 7, 8 and 9 it can clearly be seen that by the increase of the purposed schemes from R_1/1_ to R_2/3_ and from R_2/3_ to R_3/3_ for error, the value of ARL also increases which shows that the run rule scheme is not preferred for accounting variability in a process which is due to the fact that the EWMA charts are insensitive to changes in variability around the regression line stated by Kang and Albin^14^ and by Kim et al.^15^. The same case arises for the DEWMA3 chart as well. There is a good point by considering the error variance in the study, which is the use of CVRL measure, computed for each of the scheme showing consistent results than the previous run rule scheme i.e., R_3/3_ is more consistent than R_2/3_ and R_2/3_ is consistent than R_1/1_ for error variance.

The standard deviation run length or SDRL has shown inverse relation with the level of shifts “Ω_a_”, “Ω_b”_ and “Ω_e”._ It can be observed from all the Tables 1, 2, 3, 4, 5, 6, 7, 8 and 9 that the value of SDRL decreases by the increasing values of the shift levels. It can also be seen that the decreasing trend still prevails even when the run rules are increased.

Coefficient of variation of run length or CVRL were also computed for each of the proposed schemes and shown in Tables 1, 2, 3, 4, 5, 6, 7, 8 and 9 which states the amount of risk involved in selecting the scheme. Results showed that $\lambda$ = 0.1 is working significantly better in all the proposed run rule schemes than the $\lambda$ = 0.2. Whereas $\lambda$ = 0.2 is working better than the existing DEWMA3 scheme by detecting the problem more quickly.

In Tables 1, 2, 3, 4, 5, 6, 7, 8 and 9, behavior of median of run length kept on fluctuating in all the proposed run rule methods. It gives an idea about the asymmetry of the data as well as the indication about outliers. Median Deviation Run Length (MDRL) produces the same result when the value of shift level is increased till the maximum value of shift level. It could be observed that as the run rules increased from R_1/1_, R_2/3_ and R_3/3_ MDRL did not show much difference.

It is observed that all the proposed schemes under step shifts in Ω_a,_ Ω_b_ and Ω_e_ with $\lambda$=0.1 identifies the out-of-control signals very quickly than $\lambda$=0.2 showing that the lesser amount of smoothing constant has greater ability in identifying the false alarm rate, hence yielding better results (cf. Figures 1, 2, 3, 4, 5 for λ = 0.2 only).

In Figs. 1 and 2 the best among all the proposed schemes i.e., R_2/3_ with $\lambda$=0.2 is compared with the existing scheme proposed by Abdella et al.^51^ with the same value of smoothing constant. The reported results here for λ = 0.2 showed the excellency of our proposed scheme against existing scheme in terms of ARL comparison measure. In Figs. 3, 4 and 5, we used CVRL measure for checking the best proposed scheme having high level of consistency. These figures showed that the proposed scheme R_3/3_ is the most consistent one, while checking for variation in Ω_a,_ Ω_b_ and Ω_e_ with $\lambda$ = 0.2 is the most consistent one scheme.

## Case study

This section provides the real data application for linear profile charting with the implementation of runs rules schemes under simple model. This study considered the data set from the non-isothermal continuous stirred tank chemical reactor model named as CSTR process^33^. A continuous stirred tank chemical reactor (CSTR) is a commonly used reactor in the chemical industry. It is a well-mixed reactor where the reactants are continuously fed into the reactor and the products are continuously removed^52^. In a non-isothermal CSTR, the temperature inside the reactor is not constant and changes with time. A mathematical model comprising nine variables can be developed to describe the behavior of a non-isothermal CSTR.

This study selected two variables such as: the variable *X* as Inlet Concentration of Solvent Flow (CAS in K mol/m^3^) and *Y* as the Outlet Concentration of the Product (CA in K mol/m^3^). The descriptive statistics about the two associated variable is provided in Table 10. There are *N* = 1024 total observations for each variable measured over the interval of 30 s and these observations are further divided into 102 profiles with sample profiles size of 10. The first 51 profiles are considered under in-control state that are further used to estimate the lower and upper control limits of DEWMA charts under R_1/1_ and R_3/3_ run rule schemes. Further, the implementation is developed with the following steps:

**Table 10:** Descriptive statistics of CAS (inlet concentration of solvent flow) and CA (outlet concentration of the product).

CAS		CA	
Mean	0.1008	Mean	0.7998
Median	0.1029	Median	0.7958
Standard deviation	0.0490	Standard deviation	0.0514
Kurtosis	0.2184	Kurtosis	0.2315
Skewness	–0.1260	Skewness	0.2327
Range	0.3423	Range	0.3619
Minimum	−0.0682	Minimum	0.6373
Maximum	0.2740	Maximum	0.9992
Q_1_	0.0698	Q_1_	0.7673
Q_3_	0.1336	Q_3_	0.8335
W statistic (Shapiro–Wilk normality test)	0.9974	W statistic (Shapiro–Wilk normality test)	0.9953
p-value	0.1037	p-value	0.0630

Step I: For the IC regression model, following estimated model are obtained from the first 512 sample values of CA against CAS values.

$$CA=0.7942+0.0891CAS$$

$$S.E=\left(0.0056\right); (0.0491)$$

Step II: For the analysis purpose, the standard deviation of CA is calculated $\left(\widehat{\sigma }=0.0514\right)$, the smoothing parameter is fixed at 0.2 (λ = 0.2), sample size is considered as four (*n* = *4*). To obtain a charting constant of DEWMA charts under R_1/1_ and R_3/3_ are fixed at ARL_0_ = 200.

Step III: The control limits of DEWMA control charts under R_1/1_ are estimated i.e., for intercept as (0.4760, 1.1124), for slope as (−0.7781, 0.9563) and for error variance as (0.0, 0.70), while the control limits under R_3/3_ run rule are estimated such as: for intercept (0.5887, 0.9997), for slope (−0.4683, 0.6465) and for error variance (0.0, 0.4933). For this the first 51 in-control profiles are used.

Step IV: To judge the detection ability of different methodologies under run rules, variations are introduced in the data such as the shifts of 1.5, 0.15 and 1.6 are incorporated for intercept, slope, and error variance monitoring. For these the last 51 profiles are considered as out-of-control profiles.

The values of process parameters are estimated for these out-of-control profiles and then further used to compute the DEWMA statistics under R_1/1_ and R_3/3_ run rules. The charting statistics are plotted against the control limits computed in step III above. For the case of shift in intercept, the first out-of-control point occurs at 53rd profile when DEWMA statistic is 1.0483 under R_3/3_ run rule, while for R_1/1_ run rule the first out-of-control occur at 60th profile when the value of DEWMA statistic is 0.3737. On similar lines the DEWMA statistics of intercept, slope, and error variance are computed for all profiles and plotted against the respective control limits under R_1/1_ and R_3/3_ run rules.

**Figure 7:**
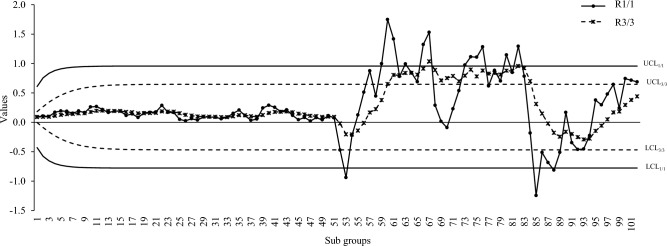
DEWMA chart display under R_1/1_ and R_3/3_ run rule scheme for shift in slope.

**Figure 8:**
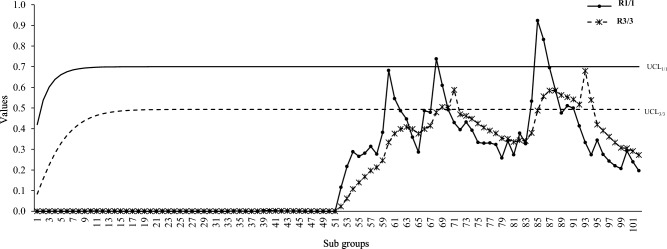
DEWMA chart display under R_1/1_ and R_3/3_ run rule scheme for shift in errors variance.

Figures 6, 7 and 8 shows the run rule scheme of R_3/3_ comes up with more out-of-control points compared to simple R_1/1_ scheme such as: the R_3/3_ scheme shows 7, 10, 9 more out-of-control points compared to R_1/1_ for intercept, slope and errors variance shifts respectively. The above findings are in accordance with the simulation study which concludes that run rule schemes are effective to improve the detection potentials DEWMA charts for linear profiles monitoring.

## Conclusion

We used various performance assessment measures such as ARLs, SDRLs, MDRLs and CVRLs on the proposed methods. The ARLs of the proposed schemes are compared with the ARL of existing study of Abdella et al.^51^. We also made an addition by considering the CVRL as well for checking the consistency of the proposed schemes. Our proposed control charts outperform than the existing control charts. Malfunctioning of the procedure will be identified by using this for upholding the quality of product and to favor both producer and client or consumer.

The proposed study scheme for given control chart is good to observe the small change or moderate change in the system and it is seen that by taking smaller value of smoothing constant, results turn to give more ideal results by detecting the problem more quickly. Run Rules provide superior results for considering intercept and slope. It is also observed that like EWMA charts DEWMA charts are also insensitive to small changes in the shift level. By using the CVRL measure, we come to know that consistency increases by increasing the run rule scheme. Researchers can also explore run rule scheme for multivariate and non-linear profiles. The run rule schemes for the monitoring of Bayesian linear profiles as well.

## Data Availability

All data generated or analyzed during this study are included in this published article^33^.
